# Supplementary material for: End Sequence Analysis Toolkit (ESAT) expands the extractable information from single-cell RNA-seq data
Source: Genome Res. 2016 Oct;26(10):1397–410. doi: 10.1101/gr.207902.116 (PMC5052061; doi:10.1101/gr.207902.116)
Supplement: Supplemental Material [file supp_gr.207902.116_Supp_Materials.tar.gz › ESAT-esat_v0.1/doc/html/classbroad_1_1core_1_1math_1_1_empirical_distribution.html]

ESAT: broad.core.math.EmpiricalDistribution Class Reference


ESAT


- Main Page
- Related Pages
- Packages
- Classes
- Files

- Class List
- Class Index
- Class Hierarchy
- Class Members

All Classes Namespaces Files Functions Variables Enumerator Pages

- broad
- core
- math
- EmpiricalDistribution

Public Member Functions |
Static Public Member Functions |
Static Public Attributes |
List of all members

broad.core.math.EmpiricalDistribution Class Reference

Collaboration diagram for broad.core.math.EmpiricalDistribution:

[legend]

|  |  |
| --- | --- |
| Public Member Functions | |
|  | EmpiricalDistribution (int numberOfBins, double min, double max) |
|  | |
|  | EmpiricalDistribution (int numberOfBins, double min, double max, boolean includeOutOfRange) |
|  | |
|  | EmpiricalDistribution (Collection< Double > vals, int numberOfBins) |
|  | |
| double | getMax () |
|  | |
| double | getMin () |
|  | |
| int | getBinNumber () |
|  | |
|  | EmpiricalDistribution (Collection< Double > vals) |
|  | |
|  | EmpiricalDistribution (Collection< Double > vals, int numberOfBins, boolean absVal) |
|  | |
|  | EmpiricalDistribution (double[] vals, int numberOfBins) |
|  | |
|  | EmpiricalDistribution (List< Double > vals, int numberOfBins) |
|  | |
|  | EmpiricalDistribution (double[] vals, int numberOfBins, double min, double max) |
|  | |
| double | testGoodnessOfFit (EmpiricalDistribution other) throws IllegalArgumentException |
|  | |
| double | chiSquareDistance (EmpiricalDistribution other) throws IllegalArgumentException, IOException |
|  | |
| double | KLDivergence (EmpiricalDistribution other) throws IllegalArgumentException |
|  | |
| double | KLDivergenceSym (EmpiricalDistribution other) throws IllegalArgumentException, IOException |
|  | |
|  | EmpiricalDistribution (InputStream is) throws IOException |
|  | |
|  | EmpiricalDistribution (File source) throws IOException |
|  | |
|  | EmpiricalDistribution (Matrix permutations, int numberOfBins) |
|  | |
| void | add (double observation) |
|  | |
| double | leftIntersect (EmpiricalDistribution other) |
|  | |
| int | getBin (double value) |
|  | |
| double | getBinMidPoint (int bin) |
|  | |
| double | getBinStart (int bin) |
|  | |
| double | getBinEnd (int bin) |
|  | |
| double | rightIntersect (EmpiricalDistribution other) |
|  | |
| double | getFirstIntersectLeftFromMedian (EmpiricalDistribution other) |
|  | |
| double | getFirstIntersectRightFromMedian (EmpiricalDistribution other) |
|  | |
| int | getTotalObservations () |
|  | |
| void | ensureNoEmptyBins () |
|  | |
| void | addPsuedocounts (double psuedocount) |
|  | |
| double | getDensity (int bin) |
|  | |
| double | getHistogram (int bin) |
|  | |
| EmpiricalDistribution | getDistributionOfHistogramValues () |
|  | |
| double | getRawDensity (int bin) |
|  | |
| double | getPercentOfValuesBetween (double leftBound, double rightBound) |
|  | |
| double | getPercentOfValuesLargerThan (double value) |
|  | |
| double | getMean () |
|  | |
| double | getStandardDeviation () |
|  | |
| double | getMedianOfAllDataValues () |
|  | |
| double | getQuantileOfBins (double probability) |
|  | |
| double | getCumulativeProbability (double value) |
|  | |
| int | getNumberOfValuesLargerThan (double value) |
|  | |
| double | getKSStatistic (List< Double > observed) |
|  | |
| double | getKSStatistic (double[] observed) |
|  | |
| void | write (BufferedWriter bw) throws IOException |
|  | |
| void | write (String save) throws IOException |
|  | |
| void | addDistribution (EmpiricalDistribution other) |
|  | |
| Collection< Double > | getAllDataValues () |
|  | |
| void | addAll (Collection< Double > values) |
|  | |
| void | addAll (double[] values) |
|  | |
| void | addAll (Matrix vals) |
|  | |
| Map< Double, Double > | getCountByBinStart () |
|  | |
| double | getProbability (double size) |
|  | |
| double | getPValue (double score) |
|  | |
| double | getPValue (double score, double min) |
|  | |
| int | numBins () |
|  | |
| double | getPValue2 (double score) |
|  | |
| double | getZscore (double value) |
|  | |

|  |  |
| --- | --- |
| Static Public Member Functions | |
| static void | main (String[] args) throws Exception |
|  | |
| static EmpiricalDistribution | getEmpiricalMaxStatisticDistribution (Collection< EmpiricalDistribution > distributions) |
|  | |

|  |  |
| --- | --- |
| Static Public Attributes | |
| static final String | USAGE |
|  | |

## Constructor & Destructor Documentation

|  |  |  |  |
| --- | --- | --- | --- |
| broad.core.math.EmpiricalDistribution.EmpiricalDistribution | ( | int | *numberOfBins*, |
|  |  | double | *min*, |
|  |  | double | *max* |
|  | ) |  |  |

Here is the caller graph for this function:

|  |  |  |  |
| --- | --- | --- | --- |
| broad.core.math.EmpiricalDistribution.EmpiricalDistribution | ( | int | *numberOfBins*, |
|  |  | double | *min*, |
|  |  | double | *max*, |
|  |  | boolean | *includeOutOfRange* |
|  | ) |  |  |

|  |  |  |  |
| --- | --- | --- | --- |
| broad.core.math.EmpiricalDistribution.EmpiricalDistribution | ( | Collection< Double > | *vals*, |
|  |  | int | *numberOfBins* |
|  | ) |  |  |

Here is the call graph for this function:

|  |  |  |  |  |  |
| --- | --- | --- | --- | --- | --- |
| broad.core.math.EmpiricalDistribution.EmpiricalDistribution | ( | Collection< Double > | *vals* | ) |  |

|  |  |  |  |
| --- | --- | --- | --- |
| broad.core.math.EmpiricalDistribution.EmpiricalDistribution | ( | Collection< Double > | *vals*, |
|  |  | int | *numberOfBins*, |
|  |  | boolean | *absVal* |
|  | ) |  |  |

Here is the call graph for this function:

|  |  |  |  |
| --- | --- | --- | --- |
| broad.core.math.EmpiricalDistribution.EmpiricalDistribution | ( | double[] | *vals*, |
|  |  | int | *numberOfBins* |
|  | ) |  |  |

Here is the call graph for this function:

|  |  |  |  |
| --- | --- | --- | --- |
| broad.core.math.EmpiricalDistribution.EmpiricalDistribution | ( | List< Double > | *vals*, |
|  |  | int | *numberOfBins* |
|  | ) |  |  |

Here is the call graph for this function:

|  |  |  |  |
| --- | --- | --- | --- |
| broad.core.math.EmpiricalDistribution.EmpiricalDistribution | ( | double[] | *vals*, |
|  |  | int | *numberOfBins*, |
|  |  | double | *min*, |
|  |  | double | *max* |
|  | ) |  |  |

Here is the call graph for this function:

|  |  |  |  |  |  |
| --- | --- | --- | --- | --- | --- |
| broad.core.math.EmpiricalDistribution.EmpiricalDistribution | ( | InputStream | *is* | ) | throws IOException |

|  |  |  |  |  |  |
| --- | --- | --- | --- | --- | --- |
| broad.core.math.EmpiricalDistribution.EmpiricalDistribution | ( | File | *source* | ) | throws IOException |

|  |  |  |  |
| --- | --- | --- | --- |
| broad.core.math.EmpiricalDistribution.EmpiricalDistribution | ( | Matrix | *permutations*, |
|  |  | int | *numberOfBins* |
|  | ) |  |  |

Here is the call graph for this function:

## Member Function Documentation

|  |  |  |  |  |  |
| --- | --- | --- | --- | --- | --- |
| void broad.core.math.EmpiricalDistribution.add | ( | double | *observation* | ) |  |

Here is the caller graph for this function:

|  |  |  |  |  |  |
| --- | --- | --- | --- | --- | --- |
| void broad.core.math.EmpiricalDistribution.addAll | ( | Collection< Double > | *values* | ) |  |

Here is the call graph for this function:

Here is the caller graph for this function:

|  |  |  |  |  |  |
| --- | --- | --- | --- | --- | --- |
| void broad.core.math.EmpiricalDistribution.addAll | ( | double[] | *values* | ) |  |

Here is the call graph for this function:

|  |  |  |  |  |  |
| --- | --- | --- | --- | --- | --- |
| void broad.core.math.EmpiricalDistribution.addAll | ( | Matrix | *vals* | ) |  |

Here is the call graph for this function:

|  |  |  |  |  |  |
| --- | --- | --- | --- | --- | --- |
| void broad.core.math.EmpiricalDistribution.addDistribution | ( | EmpiricalDistribution | *other* | ) |  |

|  |  |  |  |  |  |
| --- | --- | --- | --- | --- | --- |
| void broad.core.math.EmpiricalDistribution.addPsuedocounts | ( | double | *psuedocount* | ) |  |

|  |  |  |  |  |  |
| --- | --- | --- | --- | --- | --- |
| double broad.core.math.EmpiricalDistribution.chiSquareDistance | ( | EmpiricalDistribution | *other* | ) | throws IllegalArgumentException, IOException |

This function will calculate the chi square distance between this and other empirical distribution. A psuedocount of 1 is added to both distributions before calculating the statistic otherwise zeros will return a MathException

Author
:   skadri

Parameters
:   |  |  |
    | --- | --- |
    | other |  |

Returns

Exceptions
:   |  |  |
    | --- | --- |
    | IllegalArgumentException |  |
    | MathException |  |

|  |  |  |  |  |
| --- | --- | --- | --- | --- |
| void broad.core.math.EmpiricalDistribution.ensureNoEmptyBins | ( |  | ) |  |

|  |  |  |  |  |
| --- | --- | --- | --- | --- |
| Collection<Double> broad.core.math.EmpiricalDistribution.getAllDataValues | ( |  | ) |  |

Get list of all the data values that contributed to the bins

Returns
:   List of all data values subject to same filters as the binned counts

Here is the caller graph for this function:

|  |  |  |  |  |  |
| --- | --- | --- | --- | --- | --- |
| int broad.core.math.EmpiricalDistribution.getBin | ( | double | *value* | ) |  |

|  |  |  |  |  |  |
| --- | --- | --- | --- | --- | --- |
| double broad.core.math.EmpiricalDistribution.getBinEnd | ( | int | *bin* | ) |  |

|  |  |  |  |  |  |
| --- | --- | --- | --- | --- | --- |
| double broad.core.math.EmpiricalDistribution.getBinMidPoint | ( | int | *bin* | ) |  |

|  |  |  |  |  |
| --- | --- | --- | --- | --- |
| int broad.core.math.EmpiricalDistribution.getBinNumber | ( |  | ) |  |

|  |  |  |  |  |  |
| --- | --- | --- | --- | --- | --- |
| double broad.core.math.EmpiricalDistribution.getBinStart | ( | int | *bin* | ) |  |

|  |  |  |  |  |
| --- | --- | --- | --- | --- |
| Map<Double, Double> broad.core.math.EmpiricalDistribution.getCountByBinStart | ( |  | ) |  |

Get the distribution as a map

Returns
:   Map associating bin lower bound with number of values in the bin

|  |  |  |  |  |  |
| --- | --- | --- | --- | --- | --- |
| double broad.core.math.EmpiricalDistribution.getCumulativeProbability | ( | double | *value* | ) |  |

Here is the call graph for this function:

Here is the caller graph for this function:

|  |  |  |  |  |  |
| --- | --- | --- | --- | --- | --- |
| double broad.core.math.EmpiricalDistribution.getDensity | ( | int | *bin* | ) |  |

Here is the call graph for this function:

Here is the caller graph for this function:

|  |  |  |  |  |
| --- | --- | --- | --- | --- |
| EmpiricalDistribution broad.core.math.EmpiricalDistribution.getDistributionOfHistogramValues | ( |  | ) |  |

Get distribution of number of observations per bin

Returns
:   Distribution of number of observations per bin

Here is the call graph for this function:

|  |  |  |  |  |  |
| --- | --- | --- | --- | --- | --- |
| static EmpiricalDistribution broad.core.math.EmpiricalDistribution.getEmpiricalMaxStatisticDistribution | ( | Collection< EmpiricalDistribution > | *distributions* | ) |  |

static

Here is the call graph for this function:

|  |  |  |  |  |  |
| --- | --- | --- | --- | --- | --- |
| double broad.core.math.EmpiricalDistribution.getFirstIntersectLeftFromMedian | ( | EmpiricalDistribution | *other* | ) |  |

Here is the call graph for this function:

|  |  |  |  |  |  |
| --- | --- | --- | --- | --- | --- |
| double broad.core.math.EmpiricalDistribution.getFirstIntersectRightFromMedian | ( | EmpiricalDistribution | *other* | ) |  |

Here is the call graph for this function:

|  |  |  |  |  |  |
| --- | --- | --- | --- | --- | --- |
| double broad.core.math.EmpiricalDistribution.getHistogram | ( | int | *bin* | ) |  |

Get the total number of observations in the bin

Parameters
:   |  |  |
    | --- | --- |
    | bin | Bin number |

Returns

Here is the caller graph for this function:

|  |  |  |  |  |  |
| --- | --- | --- | --- | --- | --- |
| double broad.core.math.EmpiricalDistribution.getKSStatistic | ( | List< Double > | *observed* | ) |  |

Compared a list of observed with this empirical distribution using the Kolmogorov Smirnov Test. Will be approximate since we don't store every value in this distribution.

Get signifiance with: 1 - new umontreal.iro.lecuyer.probdist.KolmogorovSmirnovDist().cdf(D, observed.size());

Parameters
:   |  |  |
    | --- | --- |
    | observed | List of observations |

Returns
:   D statistic

NOTE: UNTESTED

Here is the call graph for this function:

Here is the caller graph for this function:

|  |  |  |  |  |  |
| --- | --- | --- | --- | --- | --- |
| double broad.core.math.EmpiricalDistribution.getKSStatistic | ( | double[] | *observed* | ) |  |

Here is the call graph for this function:

|  |  |  |  |  |
| --- | --- | --- | --- | --- |
| double broad.core.math.EmpiricalDistribution.getMax | ( |  | ) |  |

|  |  |  |  |  |
| --- | --- | --- | --- | --- |
| double broad.core.math.EmpiricalDistribution.getMean | ( |  | ) |  |

Here is the call graph for this function:

Here is the caller graph for this function:

|  |  |  |  |  |
| --- | --- | --- | --- | --- |
| double broad.core.math.EmpiricalDistribution.getMedianOfAllDataValues | ( |  | ) |  |

Get median of all data values including out of range

Returns
:   Median of all individual values including out of range or -99 if no data

|  |  |  |  |  |
| --- | --- | --- | --- | --- |
| double broad.core.math.EmpiricalDistribution.getMin | ( |  | ) |  |

|  |  |  |  |  |  |
| --- | --- | --- | --- | --- | --- |
| int broad.core.math.EmpiricalDistribution.getNumberOfValuesLargerThan | ( | double | *value* | ) |  |

|  |  |  |  |
| --- | --- | --- | --- |
| double broad.core.math.EmpiricalDistribution.getPercentOfValuesBetween | ( | double | *leftBound*, |
|  |  | double | *rightBound* |
|  | ) |  |  |

Here is the call graph for this function:

|  |  |  |  |  |  |
| --- | --- | --- | --- | --- | --- |
| double broad.core.math.EmpiricalDistribution.getPercentOfValuesLargerThan | ( | double | *value* | ) |  |

Here is the call graph for this function:

|  |  |  |  |  |  |
| --- | --- | --- | --- | --- | --- |
| double broad.core.math.EmpiricalDistribution.getProbability | ( | double | *size* | ) |  |

Here is the call graph for this function:

|  |  |  |  |  |  |
| --- | --- | --- | --- | --- | --- |
| double broad.core.math.EmpiricalDistribution.getPValue | ( | double | *score* | ) |  |

|  |  |  |  |
| --- | --- | --- | --- |
| double broad.core.math.EmpiricalDistribution.getPValue | ( | double | *score*, |
|  |  | double | *min* |
|  | ) |  |  |

|  |  |  |  |  |  |
| --- | --- | --- | --- | --- | --- |
| double broad.core.math.EmpiricalDistribution.getPValue2 | ( | double | *score* | ) |  |

P(x>Score)

Parameters
:   |  |  |
    | --- | --- |
    | score |  |

Returns

Here is the call graph for this function:

|  |  |  |  |  |  |
| --- | --- | --- | --- | --- | --- |
| double broad.core.math.EmpiricalDistribution.getQuantileOfBins | ( | double | *probability* | ) |  |

Here is the call graph for this function:

|  |  |  |  |  |  |
| --- | --- | --- | --- | --- | --- |
| double broad.core.math.EmpiricalDistribution.getRawDensity | ( | int | *bin* | ) |  |

Here is the call graph for this function:

Here is the caller graph for this function:

|  |  |  |  |  |
| --- | --- | --- | --- | --- |
| double broad.core.math.EmpiricalDistribution.getStandardDeviation | ( |  | ) |  |

Here is the call graph for this function:

|  |  |  |  |  |
| --- | --- | --- | --- | --- |
| int broad.core.math.EmpiricalDistribution.getTotalObservations | ( |  | ) |  |

Here is the caller graph for this function:

|  |  |  |  |  |  |
| --- | --- | --- | --- | --- | --- |
| double broad.core.math.EmpiricalDistribution.getZscore | ( | double | *value* | ) |  |

|  |  |  |  |  |  |
| --- | --- | --- | --- | --- | --- |
| double broad.core.math.EmpiricalDistribution.KLDivergence | ( | EmpiricalDistribution | *other* | ) | throws IllegalArgumentException |

This function will calculate the KULLBACK-LIEBLER DIVERGENCE between this and other empirical distribution. MAKE SURE THAT GIBB'S INEQUALITY HOLDS. FOR THIS, ADD 1.0 AS PSUEDOCOUNTS (USE addPsuedocounts() FUNCTION)

Parameters
:   |  |  |
    | --- | --- |
    | other |  |

Returns

Exceptions
:   |  |  |
    | --- | --- |
    | IllegalArgumentException |  |
    | MathException |  |

Here is the call graph for this function:

|  |  |  |  |  |  |
| --- | --- | --- | --- | --- | --- |
| double broad.core.math.EmpiricalDistribution.KLDivergenceSym | ( | EmpiricalDistribution | *other* | ) | throws IllegalArgumentException, IOException |

|  |  |  |  |  |  |
| --- | --- | --- | --- | --- | --- |
| double broad.core.math.EmpiricalDistribution.leftIntersect | ( | EmpiricalDistribution | *other* | ) |  |

Here is the call graph for this function:

|  |  |  |  |  |  |
| --- | --- | --- | --- | --- | --- |
| static void broad.core.math.EmpiricalDistribution.main | ( | String[] | *args* | ) | throws Exception |

static

Here is the call graph for this function:

|  |  |  |  |  |
| --- | --- | --- | --- | --- |
| int broad.core.math.EmpiricalDistribution.numBins | ( |  | ) |  |

Get the number of bins

Returns
:   The number of bins

Here is the caller graph for this function:

|  |  |  |  |  |  |
| --- | --- | --- | --- | --- | --- |
| double broad.core.math.EmpiricalDistribution.rightIntersect | ( | EmpiricalDistribution | *other* | ) |  |

Here is the call graph for this function:

|  |  |  |  |  |  |
| --- | --- | --- | --- | --- | --- |
| double broad.core.math.EmpiricalDistribution.testGoodnessOfFit | ( | EmpiricalDistribution | *other* | ) | throws IllegalArgumentException |

This function will calculate the significance of the chi square statistic between this and other empirical distribution.

Parameters
:   |  |  |
    | --- | --- |
    | other |  |

Returns

Exceptions
:   |  |  |
    | --- | --- |
    | IllegalArgumentException |  |
    | MathException |  |

|  |  |  |  |  |  |
| --- | --- | --- | --- | --- | --- |
| void broad.core.math.EmpiricalDistribution.write | ( | BufferedWriter | *bw* | ) | throws IOException |

Here is the call graph for this function:

Here is the caller graph for this function:

|  |  |  |  |  |  |
| --- | --- | --- | --- | --- | --- |
| void broad.core.math.EmpiricalDistribution.write | ( | String | *save* | ) | throws IOException |

Here is the call graph for this function:

## Member Data Documentation

final String broad.core.math.EmpiricalDistribution.USAGE

static

**Initial value:**

= "Usage: EmpiricDistribution TASK=<task\_num> <task\_args>\n" +

"\tTasks:\n" +

"\t\t1. Get empirical distribution -col <column to use 0 for the first one> [-in <Input file standard input is default> -out <output file, standard output by default> -separator <tab (\t) is default>]" +

"\t\t\t -binNum <Number of bins, default is 200> -maxValue <Maximum value to consider> -minValue <Minimum value to consider>" +

"\n\t2. Combine distributions -indir <directory> -suffix <suffix of distribution files to combine> -out <output file name defalut is standard out>" +

"\n\t3. Get quantile -probability -in <Input distribution standard input is default>" +

"\n"

---

The documentation for this class was generated from the following file:

- java/broad/core/math/EmpiricalDistribution.java


---

Generated on Fri Aug 15 2014 15:10:52 for ESAT by  

 1.8.7
